# Geranylgeranylacetone Ameliorates Skin Inflammation by Regulating and Inducing Thioredoxin via the Thioredoxin Redox System

**DOI:** 10.3390/antiox12091701

**Published:** 2023-08-31

**Authors:** Tiancheng Jin, Yitong You, Wenjie Fan, Junyang Wang, Yuhao Chen, Shujing Li, Siyuan Hong, Yaxuan Wang, Ruijie Cao, Junji Yodoi, Hai Tian

**Affiliations:** 1 Department of Basic Medicine, Medical College, Shaoxing University, Shaoxing 312000, China; 2 Laboratory of Infection and Prevention, Department of Biological Response, Institute for Virus Research, Kyoto University, Kyoto 606-8507, Japan; 3 Jiaozhimei Biotechnology (Shaoxing) Co., Ltd., Shaoxing 312000, China

**Keywords:** geranylgeranylacetone, irritant contact dermatitis, thioredoxin redox system, PI3K/Akt/Nrf2 signaling pathway, anti-inflammatory effect and mechanism

## Abstract

Geranylgeranylacetone (GGA) exerts cytoprotective activity against various toxic stressors via the thioredoxin (TRX) redox system; however, its effect on skin inflammation and molecular mechanism on inducing the TRX of GGA is still unknown. We investigated the effects of GGA in a murine irritant contact dermatitis (ICD) model induced by croton oil. Both a topical application and oral administration of GGA induced TRX production and Nrf2 activation. GGA ameliorated ear swelling, neutrophil infiltration, and inhibited the expression of TNF-α, IL-1β, GM-CSF, and 8-OHdG. GGA’s cytoprotective effect was stronger orally than topically in mice. In vitro studies also showed that GGA suppressed the expression of NLRP3, TNF-α, IL-1β, and GM-CSF and scavenged ROS in PAM212 cells after phorbol myristate acetate stimulation. Moreover, GGA induced endogenous TRX production and Nrf2 nuclear translocation in PAM212 cells (dependent on the presence of ROS) and activated the PI3K-Akt signaling pathway. GGA significantly downregulated thioredoxin-interacting protein (TXNIP) levels in PAM212 cells treated with or without Nrf2 siRNA. After knocking down Nrf2 in PAM212 cells, the effect of GGA on TRX induction was significantly inhibited. This suggests that GGA suppress ICD by inducing endogenous TRX, which may be regulated by PI3K/Akt/Nrf2 mediation of the TRX redox system.

## 1. Introduction

Thioredoxin (TRX) is a small disulfide protein with redox activity in the active site sequence Cys32-Gly-Pro-Cys35 [1] and quenches reactive oxygen species (ROS) by coupling with TRX-dependent peroxidases, or peroxiredoxins. Besides its antioxidant properties, TRX has various biologic activities, such as the regulation of cellular redox process homeostasis, the activation of transcription factors, and the inhibition of intracellular apoptotic pathways. TRX plays an essential role in various inflammatory diseases, such as respiratory inflammation, digestive inflammation, dermatitis, and arthritis [2,3,4,5,6]. ROS activate phosphatidylinositol 3 kinase (PI3K), initiating the PI3K/Akt signaling pathway, which plays an important role in activating nuclear factor erythroid 2-related factor 2 (Nrf2), which regulates oxidative stress and inflammation [7]. As a transcription factor, Nrf2 balances the expression of detoxifying enzymes and antioxidant proteins, such as heme oxygenase-1 (HO-1) and TRX, in response to diverse oxidants, antioxidants, and electrophilic stimuli, in order to protect the body [8,9]. In addition, thioredoxin-interacting protein (TXNIP) is a negative regulator of TRX and binds to TRX without oxidative stress in the cell. When the intracellular ROS content increases, TRX and TXNIP are dissociated. The mechanism for this process could involve the competitive binding of ROS to TRX [10].

Geranylgeranylacetone (GGA), also known as teprenone, is a terpenoid that promotes mucin synthesis to counteract the erosion caused by gastric acid; GGA is widely used in the treatment of gastric ulcers [11,12]. GGA induces TRX mRNA and protein expression and regulates the nuclear factor kappa-light-chain-enhancer of activated B cells and activator protein-1 activation in hepatocytes [13]. GGA attenuates hydrogen peroxide-induced ROS generation in human neuroblastoma SH-SY5Y cells [14]. Hyperthermia pretreatment combined with GGA induces HO-1 [15,16], which is commonly activated by the Nrf2/antioxidant response element (ARE) signaling pathway in the presence of oxidative stress, suggesting that GGA could induce cytoprotective factors by activating the Nrf2/ARE signaling pathway. In addition, GGA attenuated the development of some conditions, such as intracerebral hemorrhage [17], retinal ischemia-reperfusion injury [18], and neuronal cell death [19], by influencing the PI3K/Akt pathway. Activation of the PI3K/Akt/Nrf2 pathway, and the release of antioxidants such as HO-1, downregulates NOD-like receptor thermal protein domain-associated protein 3 (NLRP3), thereby inhibiting the inflammatory response [20,21,22]. These findings suggest that one potential mechanism for the cytoprotective effect of GGA is the induction of TRX through regulation of the PI3K/Akt/Nrf2-mediated TRX redox system.

To test this hypothesis, we investigated the TRX-induced anti-inflammatory effect of GGA in a murine irritant contact dermatitis (ICD) model and the molecular mechanism of action for GGA in an in vitro study. The pathologic mechanism of ICD is clearly understood: exposure to various irritants exerts toxic effects on keratinocytes, which then release cytokines, which activate innate immunity [23,24,25]. Previously, we demonstrated that topically applied, TRX prevents croton oil-induced ICD by inhibiting the local formation of inflammatory cytokines and chemokines [2,26]. Nrf2 deficiency significantly increased murine ear swelling, neutrophil infiltration, and the expression of inflammatory cytokines, as well as decreased HO-1 expression. Nrf2 can inhibit ICD induced by phorbol myristate acetate (PMA), the major active component of croton oil, through a redox-dependent or redox-independent pathway [27]. These findings provide a feasible theoretic basis for our research. Our results will clarify the mechanism of the cytoprotective effect of GGA and may provide a theoretic basis for the future development of new anti-dermatitis drugs with a completely different mechanism of action to that of conventional anti-dermatitis drugs.

## 2. Materials and Methods

### 2.1. Mice

Wild-type female C57BL6 mice (8 weeks old) were purchased from Shanghai SLAC Laboratory Animal Co., Ltd. (Shanghai, China). All animals were maintained in microisolator cages and exposed to a 12 h light/12 h dark cycle, with standard feed and water ad libitum. All animal experiments were reviewed and approved by the Ethics Committee on Laboratory Animals of Shaoxing People’s Hospital (Approval No. 2023Z068).

### 2.2. ICD Model

To induce ICD, 10 μL of 2% croton oil (Sigma; St. Louis, MO, USA) dissolved in acetone/olive oil (4:1) was applied to the dorsal and ventral aspects of both sides of the murine ears. The modeling method was described in detail in our previous research [2]. Ear swelling was measured in a blinded fashion with a micrometer (Tokyo, Japan), 1, 4, 8, and 24 h after the application of croton oil.

### 2.3. Animal Treatment

Mice treated with a topical application were divided into three groups: recombinant human thioredoxin (rhTRX), GGA, and bovine serum albumin (BSA). rhTRX, GGA, and BSA were dissolved in 1% hydrogel to prepare 20 μg/mL of drug solution. An amount of 125 μL of different drugs was evenly applied to the dorsal and ventral of the ear of each group of mice, twice a day for 5 days, to gavage groups, and the same quantity of normal saline was gavaged in a control group; these three groups are defined as the gavage group. rhTRX was received as a gift from Redox Bio Science Co., Ltd. (Kyoto, Japan). GGA and hydrocortisone were purchased from Eisai Co., Ltd. (Tokyo, Japan), and BSA was purchased from Solarbio Life Sciences (Beijing, China).

### 2.4. Histologic Analysis of ICD

Croton oil was applied on Day 6, and the mice were euthanized by an intraperitoneal injection of sodium pentobarbital (100 mg/kg) 24 h later; the ears were removed and fixed in formalin for 24 h, embedded in paraffin, and stained with hematoxylin and eosin. The pathologic changes in skin tissue from each group were observed using a microscope. For the quantitative analysis, neutrophil numbers in the dermis were counted in 10 microscopic fields of five different specimens, and the average number of neutrophils was obtained.

### 2.5. Immunohistochemistry

From paraffin-embedded specimens, 4 μm thick, sectioned tissue samples were prepared and then dewaxed with 3% hydrogen peroxide methanol solution. Endogenous peroxidase activity was blocked for 15 min, and 10% BSA was added for 30 min to block nonspecific binding at room temperature. The tissue specimens were incubated with rabbit anti-mouse TNF-α polyclonal antibody (1:200; Bioss, Beijing, bs-10802R, China); rabbit anti-mouse IL-1β polyclonal antibody (1:200; Bioss bs-0812R; China); rabbit anti-mouse TRX monoclonal antibody (1:1000; Fujirebio, Tokyo, Japan); mouse anti-mouse 8-hydroxy-2’-deoxyguanosine (8-OHdG) monoclonal antibody (1:200; Santa Cruz Biotechnology, sc393871, Shanghai, China); rabbit anti-mouse GM-CSF polyclonal antibody (1:200; Bioss, bs3790R, China); rabbit anti-mouse NLRP3 polyclonal antibody (1:500; ABclonal, Wuhan, A21906, China); or mouse anti-mouse Nrf2 monoclonal antibody (1:200; Proteintech, Wuhan, 66504-1-Ig, China) as the primary antibody and incubated overnight at 4 °C. After being washed with phosphate-buffered saline (PBS), the slices were incubated with horseradish peroxidase-labeled goat anti-rabbit/murine immunoglobulin G (IgG) secondary antibody (1:50; Beyotime Biotechnology, Shanghai, A0208, China) for 30 min at room temperature, and then with 3,30-diaminobenzidine working solution (Beyotime Biotechnology, China). Quantifications were performed with Image-Pro Plus 6.0.

### 2.6. Cell Culture and Cell Processing

This study used the spontaneously transformed BALB/c keratinocyte cell line PAM 212. The cell line was provided by Dr Steve Ulik (Department of Immunology, Anderson Cancer Center, Houston, TX, USA). Cells were maintained with 10% heat-inactivated FBS (HyClone, Shanghai, China); 10 mM of Hepes (Sigma, Shanghai, China); 1% non-essential amino acids (Sigma, China); 2 mM of L-glutamine (Sigma, China); 1 mM of sodium pyruvate (Sigma, China); 100 U/mL of penicillin (Sigma, China); 100 μg/mL of streptomycin (Sigma, China); 0.25 μg/mL of amphotericin B (Sigma, China); and 2-mercaptoethanol (Sigma, China) in Roswell Park Memorial Institute 1640 medium (Sigma, China). Cells were cultured in 95% air/5% carbon dioxide at 37 °C. To evaluate the anti-inflammatory and antioxidant effects of GGA in keratinocytes, PAM212 cells in passages 1–3 were seeded into six-well plates at a density of 2 × 10^5^/2 mL medium per well and cultured for 24 h. An amount of 10 mM of PMA (Beyotime Biotechnology, China) was added to each well, followed by 1 μM of GGA, 20 μg/mL of hydrocortisone, 20 μg/mL of BSA, and 20 μg/mL of rhTRX. To further validate the role of GGA in inducing TRX through activation of the PI3K/Akt/Nrf2 signaling pathway, the PAM212 cells in each well were treated with the 50 μM of PI3K inhibitor LY294002 (Beyotime Biotechnology) or 1 mM of the antioxidant N-acetyl-L-cysteine (NAC) (Beyotime Biotechnology) for 1 h, and then incubated with 1 μM of GGA for 24 h.

### 2.7. RNA Interference of Nrf2

Nrf2 small interfering RNA (siRNA) was designed and synthesized by GenePharma (Shanghai, China). Transfection was performed using Lipofectamine™ 3000 Transfection Reagent (Thermo Fisher Scientific, Shanghai, L3000075, China), and transfection efficiency was confirmed by a real-time PCR. The siRNA sequences used in this experiment are presented in Table 1. The PAM212 cells in each well were treated with 0.5 μM of Nrf2 siRNA in 200 μL of Opti-MEM medium (Thermo Fisher Scientific, 31985070, China) and 5 μL of lipofectamine 3000 for 12 h, and then switched to the culture medium overnight. The next day, 1 μM of GGA was added for 24 h.

### 2.8. Protein Extraction and Western Blot Analysis

The cells were collected and lysed in ice-cold radioimmunoprecipitation assay buffer (Beyotime Biotechnology, Shanghai, China) containing phenylmethyl sulfonyl fluoride and phosphatase inhibitors (Solarbio, Beijing, China). The total cell lysate was centrifuged at 13,000 rpm (4 °C) for 15 min, mixed with 10 μL of 5× sample buffer (Solarbio, China), and denatured by boiling for 10 min. The protein extract was added with loading buffer (Solarbio, China) and boiled for 5 min. After cooling, each well was loaded with 30 μg of protein. Then, the proteins were separated by 12% sodium dodecyl-sulfate polyacrylamide gel electrophoresis and transferred to a polyvinylidene fluoride membrane by electrophoresis. The membrane was blocked in tris-buffered saline with Tween 20 (TBST) with BSA 5% for 2 h and incubated overnight with primary antibodies TNF-α (1:1000), IL-1β (1:1000), GM-CSF (1:1000), TRX (1:10,000), TXNIP (1:1500; Affinity Biosciences, Jiangsu, DF7506, China), and Nrf2 (1:10,000; Affinity Biosciences, DF7506, China) at 4 °C. After three washes with TBST, the membrane was incubated with horseradish peroxidase-labeled goat anti-rabbit/murine IgG secondary antibody (1:1000; Beyotime Biotechnology, A0208, China) at 37 °C for 2 h. After washing in TBST three times, the protein bands were detected using an enhanced chemiluminescence detection system. A density analysis was performed using ImageJ 1.47v (National Institutes of Health, Bethesda, MD, USA).

### 2.9. Flow Cytometry Assays for ROS Production

To assess intracellular ROS levels, PAM212 cells were seeded into a 24-well plate at a density of 1 × 10^5^/500 μL medium per well and cultured for 24 h. An amount of 10 nM of PMA was added to the medium in the presence or absence of 1 μM of GGA or 1 mM of NAC for 6 h; then, the cells were incubated with 10 μmol/L of dichlorodihydrofluorescein diacetate (DCF-DA) for 30 min at 37 °C and washed twice in PBS. Then, DCF-DA staining was performed. Images were acquired using a Leica 300 fluorescence microscope (Leica Microsystems, Wetzlar, Germany), and green fluorescence was detected using flow cytometry (Beckman Coulter, Brea, CA, USA). At least 10,000 cells were collected per sample and data were analyzed using CytExpert 2.4 software (Beckman Coulter).

### 2.10. Real-Time Reverse Transcription Polymerase Chain Reaction (RT-PCR) Analysis

For the RT-PCR analysis, total RNA was extracted from PAM212 cells using the Tissue/Cell RNA Rapid Extraction Kit (Aidlab Biotechnologies, Ltd., Beijing, China). On ice, RNA was converted to cDNA by reverse transcription using a reverse transcription kit (Bio-Rad Laboratories Inc., Hercules, CA, USA). To quantify RNA, relative mRNA expression levels were assessed by RT-PCR using the PowerUp™ SYBR™ Green system (Thermo Fisher Scientific, A25742, China) and the LightCycler 96 System (Roche, Germany). Using the 2^−ΔΔCt^ method, the cycle time values for each sample were normalized to glyceraldehyde 3-phosphate dehydrogenase (B661304-0001; Sangon Biotech, Shanghai, China). The primers used were purchased from Sangon Biotech, and the primer sequences are presented in Table 1.

### 2.11. Statistical Analyses

Statistical analyses were performed using GraphPad Prism 9.3.1 software (La Jolla, CA, USA). The Student’s *t*-test was used to compare means for two groups. Where appropriate, data from multiple groups were analyzed using a one-way analysis of variance (ANOVA), or a two-way ANOVA with post hoc Tukey’s. The results were presented as mean ± standard (SD). Significance levels were set at probabilities of *p* < 0.05 (*), *p* < 0.01 (**), *p* < 0.001 (***), and *p* < 0.0001 (****).

## 3. Results

### 3.1. Induction of TRX and Nrf2 Expression by GGA in ICD Induced by Croton Oil

**Figure 1:**
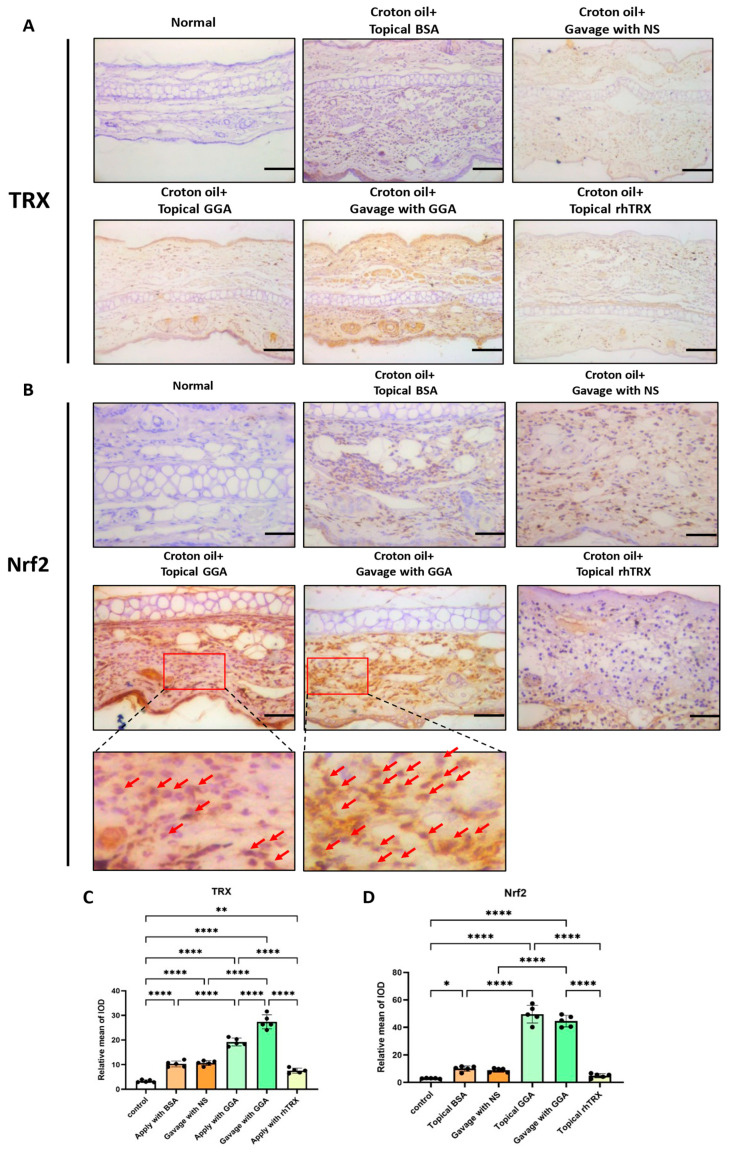
Induction of TRX and Nrf2 by GGA in the ICD model. (**A**,**B**) Immunohistochemistry was performed to detect the effects of GGA on TRX induction and Nrf2 activation. GGA induces the production of TRX, especially after oral administration. Normal = mice without any treatment. NS = normal saline; bars 100 μm. The most pronounced nuclear translocation of Nrf2 was in the GGA gavage-treated group, followed by the GGA topically applied group; bars 50 μm; n = 5 mice for each group. Cells with nuclear translocation are indicated by arrows. (**C**,**D**) The mean of IOD expression of TRX and Nrf2. Values are shown as the mean ± SD (* *p* < 0.05, ** *p* < 0.01, **** *p* < 0.0001; one-way ANOVA with post hoc Tukey’s).

Compared to the group without croton oil application, due to inflammatory stimulation, a small amount of TRX was present in tissues in the BSA-applied group and the normal saline-gavaged group. In the GGA-applied group, TRX expression was enhanced, especially in the epidermis. In the GGA-gavage group, TRX was markedly distributed in both the dermis and epidermis. TRX expression in the rhTRX-applied group was similar to that in the BSA-applied group and normal saline-gavaged group under inflammatory conditions (Figure 1). These findings indicate that endogenous TRX is induced by the topical application of GGA, and especially by the oral administration of GGA. Meanwhile, Nrf2 expression was increased by GGA (vs. control) in murine ear tissue in the ICD model. Nuclear Nrf2 expression was greater in the GGA-gavaged group than in the GGA topically applied group (Figure 1). This suggests that GGA facilitated Nrf2 translocation from the cytoplasm to the nucleus in the ICD model.

### 3.2. GGA Suppressed ICD Induced by Croton Oil

**Figure 2:**
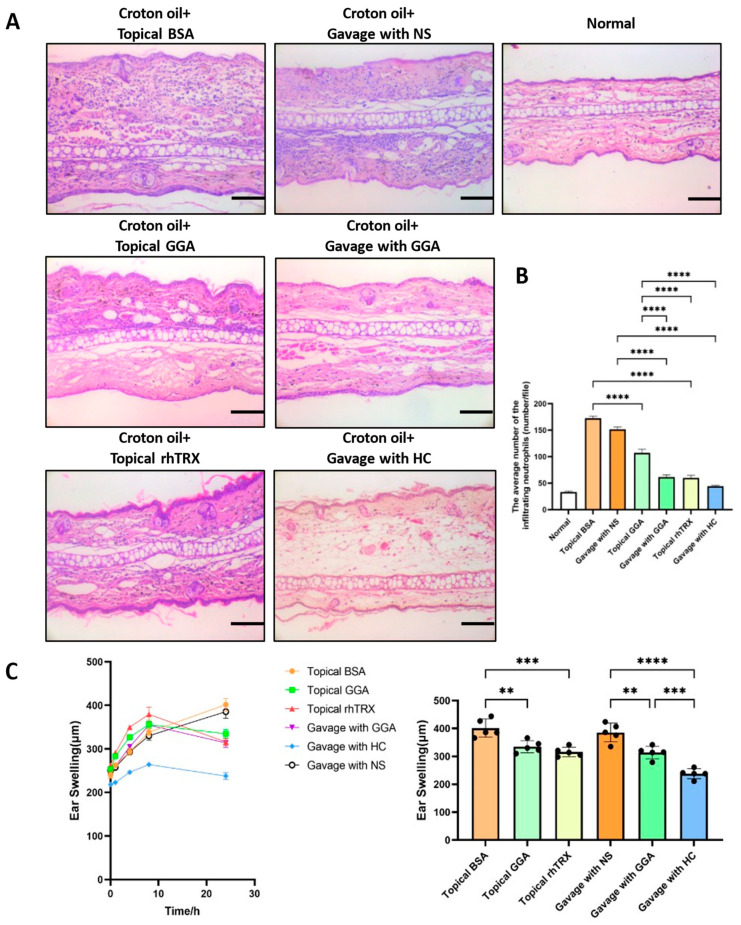
The anti-inflammatory effect of GGA in the ICD model. (**A**) Representative pictures of hematoxylin and eosin staining of the ears. Pretreatment with GGA, rhTRX, and hydrocortisone (HC) inhibited skin inflammation, such as neutrophil infiltration and edema, compared to the negative control group; bars 100 μm. (**B**) The mean number of dermal neutrophil infiltrates was significantly reduced in the GGA and rhTRX topically applied groups, and in the GGA and HC gavage-treated groups, compared to the negative control group (**** *p* < 0.0001); n = 5 mice for each group; values are mean ± SD (one-way ANOVA with post hoc Tukey’s). (**C**) Ear thickness changes in croton oil-treated mice treated with GGA, rhTRX, HC, BSA, and NS (** *p* < 0.01, *** *p* < 0.001, **** *p* < 0.0001); n = 5 mice for each group; values are mean ± SD (one-way ANOVA with post hoc Tukey’s).

To examine the anti-inflammatory effect of GGA on ICD induced by croton oil, a histologic analysis was performed. Both a topical application and oral administration of GGA inhibited auricular inflammation, such as edema and the infiltration of inflammatory cells, including neutrophils and macrophages, compared to the BSA-applied group and the normal saline (NS)-gavaged group. Further, topically applied rhTRX also suppressed skin inflammation, and these results are consistent with our previous report. As expected, hydrocortisone had a good anti-inflammatory effect (Figure 2A). In addition, the numbers of tissue neutrophils were counted and measured to evaluate the anti-inflammatory effects in each group. Mean neutrophil infiltration numbers were significantly reduced in each group after croton oil stimulation, except in the BSA-applied group and the NS-gavaged group. Among these groups, the anti-inflammatory effect was more marked after orally administered GGA, topically applied rhTRX, and orally administered hydrocortisone, compared to topically applied GGA (*p* < 0.0001; Figure 2B). The inflammatory response was expressed as an average increase in ear swelling. Treatment with GGA (*p* < 0.01), rhTRX (*p* < 0.001), and hydrocortisone (*p* < 0.0001) significantly suppressed ear swelling 24 h after croton oil application (Figure 2C). These findings indicate that both topically applied GGA and orally administrated GGA inhibited ICD.

### 3.3. GGA Inhibited TNF-α, IL-1β, GM-CSF, and 8-OHdG Expression in ICD Induced by Croton Oil

**Figure 3:**
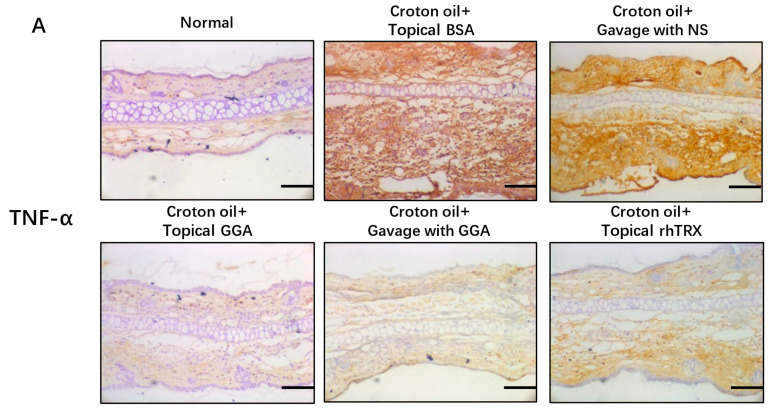

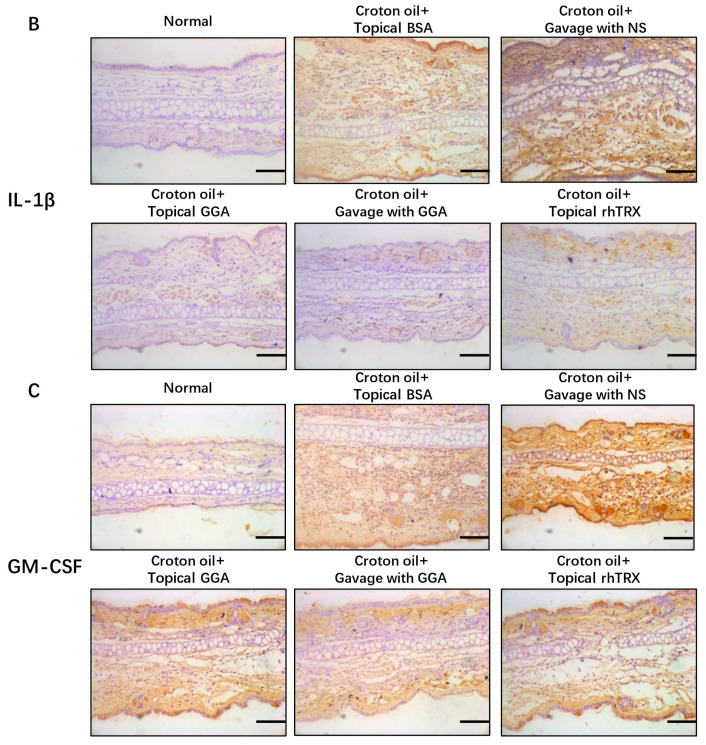

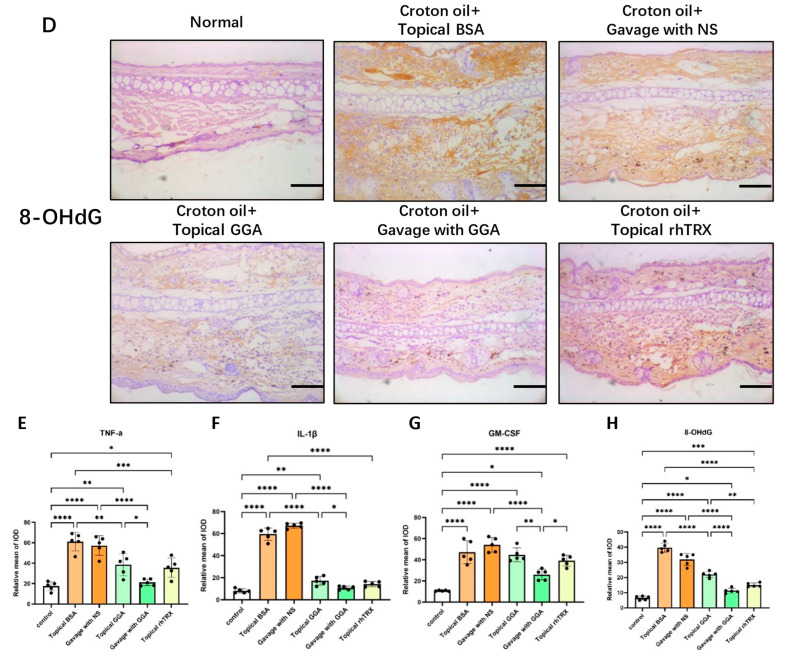
Topical and oral treatment with GGA inhibited the expression of TNF-α, IL-1β, GM-CSF, and 8-OHdG in a croton oil-induced ICD model. (**A**–**D**) Immunohistochemistry was performed to detect the effects of GGA on cytokine production and antioxidants. Immunohistochemical staining showed that the expression of TNF-α, IL-1β, GM-CSF, and 8-OHdG was strongly inhibited by GGA; bars 100 μm; n = 5 mice for each group. (**E**–**H**) The mean of IOD expression of TNF-α, IL-1β, GM-CSF, and 8-OHdG. Values are shown as the mean ± SD (* *p* < 0.05, ** *p* < 0.01, *** *p* < 0.001, **** *p* < 0.0001; one-way ANOVA with post hoc Tukey’s).

The expression of selected cytokines in murine ear tissue was studied by immunohistochemical staining 24 h after the addition of croton oil. In negative controls, TNF-α, IL-1β, GM-CSF, and 8-OHdG were strongly induced and widely expressed in the dermis and epidermis. However, cytokine expression was inhibited after topically applied GGA, orally administered GGA, and topically applied rhTRX; gavaged GGA was superior to topically applied GGA and topically applied rhTRX (Figure 3). The results indicate that both topically applied and orally administered GGA inhibited inflammatory cytokine production and oxidative stress in the ICD model.

### 3.4. GGA Inhibited PMA-Induced TNF-α, IL-1β, GM-CSF, and NLRP3 Expression in Murine Keratinocytes

**Figure 4:**
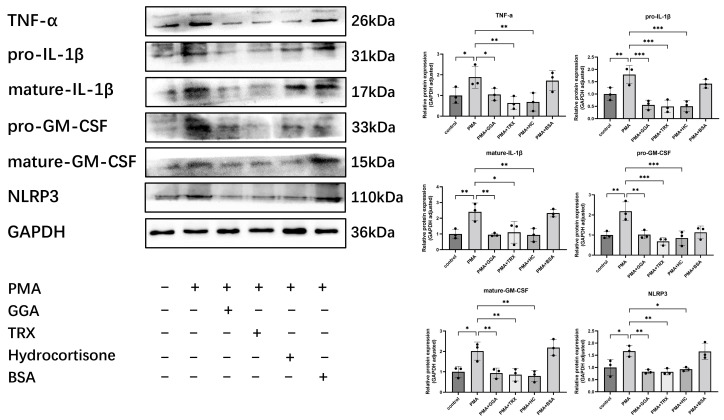
GGA significantly inhibited the protein expression of TNF-α, IL-1β, GM-CSF, and NLRP3 in PMA-stimulated PAM212 cells. An amount of 1 μM of GGA, 20 μg/mL of rhTRX, and 20 μg/mL of hydrocortisone were added to the medium in the presence of PMA. The protein expression of TNF-α, IL-1β, GM-CSF, and NLRP3 was detected by Western blot at 24 h post-stimulation. The relative expression of cytokines was quantified and normalized to GAPDH. Expression of these proteins was significantly inhibited in the GGA-treated group compared to the PMA-treated group; values are mean ± SD of three independent experiments (* *p* < 0.05, ** *p* < 0.01, *** *p* < 0.001; one-way ANOVA with post hoc Tukey’s).

The studied cytokines were diffusely expressed in the epidermis, suggesting possible cytokine production by keratinocytes in the ICD model (Figure 3). Therefore, we selected murine keratinocytes (PAM212 cells) as targets to detect the effects of GGA on cytokine production after croton oil treatment. An amount of 1 μM of GGA (*p* < 0.05), 20 μg/mL of rhTRX (*p* < 0.01), and 20 μg/mL of hydrocortisone (*p* < 0.001) all had significant inhibitory effects on the PMA-induced expression of NLRP3 and cytokines in PAM212 cells, compared with BSA + PMA (Figure 4). The results indicate that GGA inhibited PMA-induced inflammatory cytokines and NLRP3 in PAM212 cells.

### 3.5. GGA Scavenged ROS Production in PAM212 Cells Stimulated by PMA

**Figure 5:**
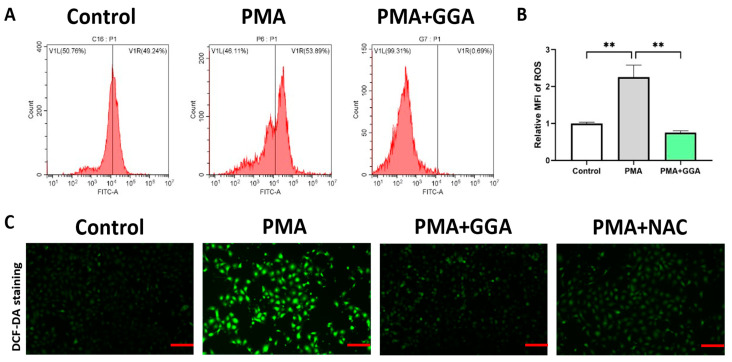
GGA inhibited ROS production in PAM212 cells treated with PMA. The ROS levels in PAM212 cells were detected by flow cytometry and DCF-DA staining after GGA or NAC was added (**A**,**C**). The MFI in flow cytometry was assessed (**B**). Values are shown as the mean ± SD of eight experiments (** *p* < 0.01; one-way ANOVA with post hoc Tukey’s). The images of DCF-DA staining were taken using a Leica 300 fluorescence microscope (ROS production, FITC, green); bars 200 µm.

GGA had a significant (*p* < 0.0001) scavenging effect on PMA-induced ROS in PAM212 cells (Figure 5A,B). The PAM212 cells stimulated by PMA produced a large amount of ROS, but intracellular ROS were eliminated by both GGA and NAC, which is a ROS scavenger. There was no difference in antioxidant effect between GGA and NAC (Figure 5C). These findings show that GGA scavenged ROS in PMA-simulated PAM212 cells.

### 3.6. Induction of TRX by GGA in PAM212 Cells Was Associated with ROS/TXNIP and PI3K/Akt/Nrf2 Signaling Pathways

**Figure 6:**
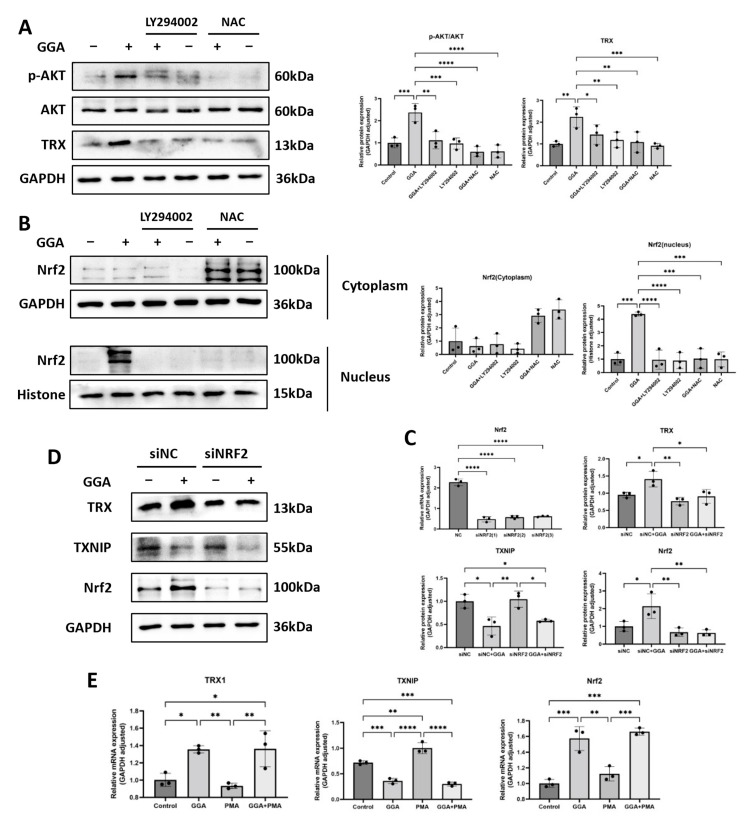
GGA induced TRX in PAM212 cells in association with the PI3K/Akt/Nrf2 signaling pathway, ROS, and TXNIP. (**A**) PAM212 cells were pretreated with 50 μM of LY294002 and 1 mM of NAC for 1 h, and then incubated with 1 μM of GGA for 24 h. The protein expression of p-Akt, Akt, and TRX was detected by Western blotting. (**B**) Intranuclear and extranuclear proteins were extracted from PAM212 cells, after the same treatment, using the Nuclear and Cytoplasmic Protein Extraction Kit (Beyotime Biotechnology), and the protein expression of Nrf2 was detected using Western blotting. (**C**) The knockdown effect of siNRF2 (1), siNRF2 (2), and siNRF2 (3) was verified using a real-time RT-PCR. (**D**) The selected PAM212 cells were transfected with siNRF2 (1) after treatment with or without 1 μM of GGA, and the protein expression of Nrf2, TRX, and TXNIP was detected using Western blotting. A grayscale analysis was performed using ImageJ 1.47v. (**E**) The mRNA expression of TRX1, TXNIP, and Nrf2 after 12 h treatment with 1 mM of PMA and 1 μM of GGA was detected using a real-time RT-PCR. Values are shown as the mean ± SD of three experiments (* *p* < 0.05, ** *p* < 0.01, *** *p* < 0.001, **** *p* < 0.0001; one-way ANOVA with post hoc Tukey’s).

The expression of p-AKT and TRX was significantly upregulated in PAM212 cells after GGA stimulation, but expression was reduced after the joint addition of LY294002 and NAC (Figure 6A). These indicate that GGA induced endogenous TRX via the PI3K-AKT signaling pathway and ROS production in PAM cells. In addition, nuclear Nrf2 levels were markedly upregulated by GGA, while no obvious changes were observed in the cytoplasm. Nuclear Nrf2 translocation was stopped by LY294002 or NAC (Figure 6B).

Nrf2 expression was knocked down by siRNA Nrf2. First, the interfering effect of three siRNA Nrf2s was rated by real-time PCR. Because siNRF2 (1) better inhibited the mRNA expression of Nrf2 in PAM212 cells, it was selected for subsequent experiments (Figure 6C). A Western blot assay revealed that GGA significantly enhanced TRX and Nrf2 expression, but the effect of GGA-induced TRX was significantly downregulated by treating with siNRF2 (1). Conversely, GGA significantly suppressed TXNIP protein level, which may have been related to ROS downregulation (Figure 6D). In addition, we detected the mRNA expression of TRX, TXNIP, and Nrf2, in PAM212 cells treated with PMA and GGA, by real-time PCR. The results were consistent with the Western blot assay (Figure 6E) and showed that GGA-induced TRX was intimately associated with Nrf2 activation and TXNIP downregulation.

In conclusion, ROS may impact the initiation of signaling pathways. GGA induced endogenous TRX by activating the PI3K/Akt/Nrf2 signaling pathway and downregulating TXNIP levels in PAM212 cells; this effect was related to ROS.

## 4. Discussion

In this study, we demonstrated that GGA induced endogenous TRX in skin tissues from murine ICD induced by croton oil. Previous studies also showed that GGA induced TRX in gastric mucosal cells (RGM-1) and peripheral blood lymphocytes [28]; therefore, GGA is a recognized TRX inducer [13,29]. In addition, TRX expression was strengthened in the orally administered versus topically applied GGA group, which may have been due to the skin barrier hindering the penetration of topically applied GGA into the deep layers, thereby affecting TRX induction; however, orally administered GGA could enter skin tissue via the circulation to better exert a TRX-inducing effect. TRX was weakly expressed in other treatment groups, potentially because of endogenous TRX produced by inflammatory stimulation. Murine anti-TRX antibodies do not cross-react with human TRX, verifying that only endogenous TRX was detected in the histologic analysis.

GGA prevented morphine-induced hyperkinesia, reward effects and withdrawal syndrome, and place preference hepatic [30,31] and renal damage induced by morphine in mice by enhancing TRX expression [32]. GGA also blocked retinal photo-oxidative damage [32], methamphetamine-induced neurotoxicity [33], and lung ischemia/reperfusion injury [34] through TRX release. However, the mechanisms underlying both the anti-inflammatory and TRX-inducing effects of GGA are unclear. This is the first study to demonstrate that GGA suppressed ICD induced by croton oil by inhibiting cytokine production and oxidative stress. The anti-inflammatory effect on rhTRX is consistent with our previous report [2]. Because TRX has excellent anti-inflammatory and antioxidant effects, as well as attenuates cytokine production, we speculate that GGA may, in part, exert its anti-inflammatory effect by inducing TRX. In addition, keratinocytes play a major role in ICD pathogenesis. Both our in vitro study and our previous study showed that cytokines were produced by keratinocytes stimulated with croton oil or PMA; the cytokines produced by PAM212 cells were also inhibited by GGA. Overall, these findings suggest that GGA blocked inflammatory reactions by inducing TRX.

It has also been reported that oxidative stress plays various important roles in contact dermatitis [35]. Intracellular ROS were increased after keratinocytes were stimulated with PMA [36]. We demonstrated that GGA inhibited 8-OhdG expression, which is an oxidative stress maker in ear tissues of ICD, and scavenged intracellular ROS in PMA-induced PAM212 cells. These findings suggest that GGA improved the oxidant/antioxidant imbalance in cutaneous tissues and cells by inducing TRX.

TRX induction could be one of the protective mechanisms of GGA, which attenuated the development of nervous system diseases by influencing the PI3K/Akt pathway. The PI3K/Akt/Nrf2 signaling pathway plays a major role in inducing TRX, and previous studies have proposed Nrf2 as a downstream target of Akt [36,37,38,39]. Moreover, Akt promotes Nrf2 nuclear translocation, which in turn binds to ARE in the TRX promoter in the form of Nrf2-Maf, in order to induce TRX expression [40]. A previous chromatin immunoprecipitation sequencing study confirmed that the use of sulforaphane in human lymphoblastoid cells resulted in Nrf2 binding to ARE in the TRX promoter [41,42]. In the present study, the PI3K/Akt/Nrf2 signaling pathway was activated in PAM212 cells after GGA treatment, but the expression of p-Akt, Nrf2, and TRX was inhibited by LY294002. These results indicate that GGA induced TRX by activating the PI3K/Akt/Nrf2 pathway.

Previous studies showed that the inducing effect of GGA on TRX had no effect after using NAC [13]. Consistently, when employing NAC to inhibit oxidative stress response, even in the presence of GGA, a significant downregulation of TRX expression levels was observed in PAM212 cells. This phenomenon arises from the role of TRX as an antioxidant protein, primarily tasked with maintaining redox equilibrium. During upregulation induced by oxidative stress, TRX expression is enhanced as a protective measure. Subsequent to the elimination of oxidative stress, TRX levels are once again downregulated to sustain the coordinated balance between oxidation and antioxidation. This observation aligns with the differential expression pattern of TRX in COPD [43]. Akt phosphorylation and the nuclear translocation of Nrf2 were also inhibited in the GGA group after NAC was applied, thus suggesting that GGA activation of the PI3K/Akt/Nrf2 pathway depended on intracellular ROS production. Therefore, we consider that ROS are one of the necessary stimuli for GGA to induce TRX via the PI3K/Akt/Nrf2 pathway.

The TRX system plays important roles in maintaining a reduced intracellular environment and in redox-dependent cell function. Under non-oxidative conditions, TXNIP binds to reduced TRX [44] and inhibits the reducing activity of TRX [45]. With intracellular oxidative stress, ROS cause TRX/TXNIP to separate. TXNIP binds to NLRP3 to induce IL-1β expression, and TRX reduces inflammation [46]. We demonstrated that GGA induced TRX and scavenged ROS in an in vivo and in vitro study. Therefore, we supposed that GGA regulated intracellular TXNIP expression. The results also indicate that GGA downregulated TXNIP in both inflammatory and non-inflammatory conditions; moreover, we found that GGA downregulated NLRP3 expression in the inflammatory state, suggesting that GGA suppressed IL-1β production by downregulating TXNIP and NLRP3 expression and ROS production. In addition, TXNIP, which is partially localized at the S308 site of the plasma membrane, was phosphorylated by Akt [47]. Further, TXNIP inhibits Akt activity [48], and the PI3K/Akt pathway is inhibited by high glucose, leading to TXNIP upregulation [49]. Forkhead box D3-antisense RNA enhanced the expression of TXNIP by inhibiting the Akt/Nrf2 pathway [50]. These results indicate that the TRX/TXNIP redox system can be regulated by the PI3K/Akt/Nrf2 signaling pathway. We demonstrated that GGA induced TRX by activating the PI3K/Akt/Nrf2 signaling pathway, which was mediated by the TRX redox system. 

Exogenous TRX activates transient receptor potential channels (TRPCs) to regulate intracellular calcium via thiol-modified redox mechanisms [51,52]. To verify the relationship between TRPCs and GGA-induced TRX, we treated PAM212 cells with SKF96365 (TRPC inhibitor) or U-73122 (phospholipase C inhibitor), together with GGA, and showed no significant change in TRX expression in the experimental versus control group (The data are shown in the Supplementary Material); the relationship between calcium and TRX may be unidirectional or more relevant in neuronal cells.

G-Rh2 passes through the FcεRI receptor on the cell membrane, which in turn activates the AKT-Nrf2 pathway [38]. We also speculate that GGA may also activate a certain receptor on the cell membrane, which in turn activates the PI3K/Akt/Nrf2 signaling pathway. In addition, it has been reported that GGA suppresses the binding of LPS to the cell surface of macrophages, resulting in inhibiting signal transduction downstream of TLR4 [53]. GGA cloud also ameliorates skin inflammation by suppressing the interaction on PMA and the cells, and then regulates the PI3K/Akt/Nrf2 signaling pathway.

## 5. Conclusions

**Figure 7:**
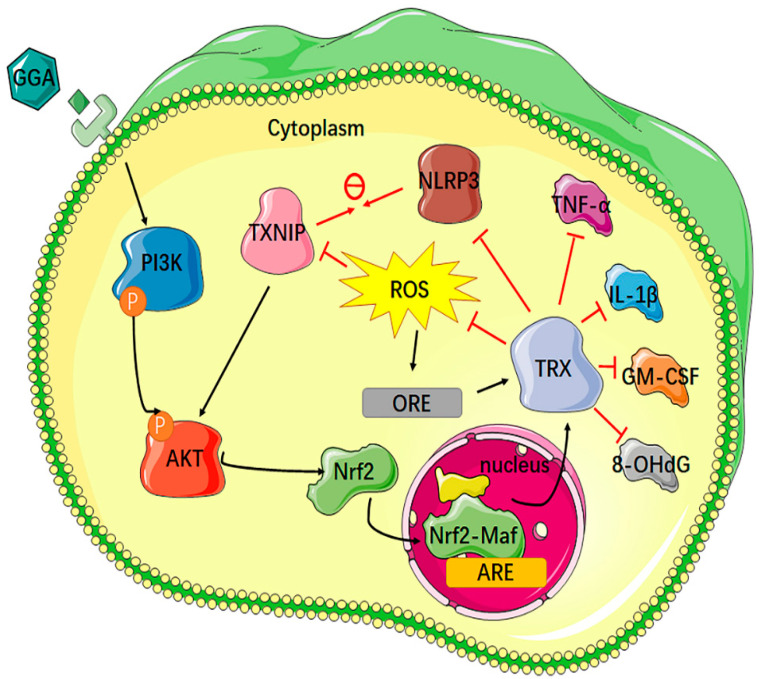
A schematic model. The induction of endogenous TRX. GGA induced TRX by enhancing Akt phosphorylation and Nrf2 translocation, thus downregulating TXNIP expression and ROS.

We demonstrated that GGA suppressed skin inflammation by inhibiting the production of cytokines, which was involved by the TRX redox system. We also further elucidated the molecular mechanism for the effect of GGA to induce TRX; this mechanism increases the possibility that GGA could be used as a new medicine to treat inflammatory diseases (Figure 7). However, we cannot confirm that GGA exerts its anti-inflammatory effect solely by inducing TRX, because GGA can also induce others, such as HO-1, nitric oxide synthase, and heat shock protein 70. We would like to refine the mechanisms involved in GGA in the future so that it is not limited to the treatment of gastric disorders.

## Figures and Tables

**Table 1:** The sequence of Nrf2 siRNA, Nrf2, TRX1 and TXNIP primers.

Name	Sequence	
	Forward	Reverse
siNRF2(1)	5′-GAGGAUGGAAGCCUUACUTT-3′	5′-AGUAAGGCUUUCCAUCCUCTC-3′
siNRF2(2)	5′-GGAGGCAAGACAUAGAUCUTT-3′	5′-AGAUCUAUGUCUUGCCUCCTT-3′
siNRF2(3)	5′-CCGAAUUACAGUGUCUUAATT-3′	5′-UUAAGACACUGUAAUUCGGTT-3′
Nrf2	5′-CAGCATGACTGATTTAAGCAG-3′	5′-CAGCTGCTTTTTCGTGTGTGTATTA-3′
TRX1	5′-TTCCCTCTGTGACAAGTTCC-3′	5′-TCAAGCTTTCTCTTGTTAGCAC-3′
TXNIP	5′-GTCTTGAGGTGGTCTTCAAC-3′	5′-TCACACACTTCCACTATTACCC-3′

## Data Availability

Data will be provided on reasonable request.

## Supplementary Materials

The following supporting information can be downloaded at: https://www.mdpi.com/article/10.3390/antiox12091701/s1.
