# Supplementary material for: Geranylgeranylacetone Ameliorates Skin Inflammation by Regulating and Inducing Thioredoxin via the Thioredoxin Redox System
Source: Antioxidants (Basel). 2023 Aug 31;12(9):1701. doi: 10.3390/antiox12091701 (PMC10525896; doi:10.3390/antiox12091701)
Supplement: Supplementary file 1 [file antioxidants-12-01701-s001.zip › antioxidants-2482568-supplementary.pdf]

Geranylgeranylacetone Ameliorates Skin Inflammation by Regulating and Inducing Thioredoxin via the Thioredoxin Redox System

Supplementary Material

Negative results

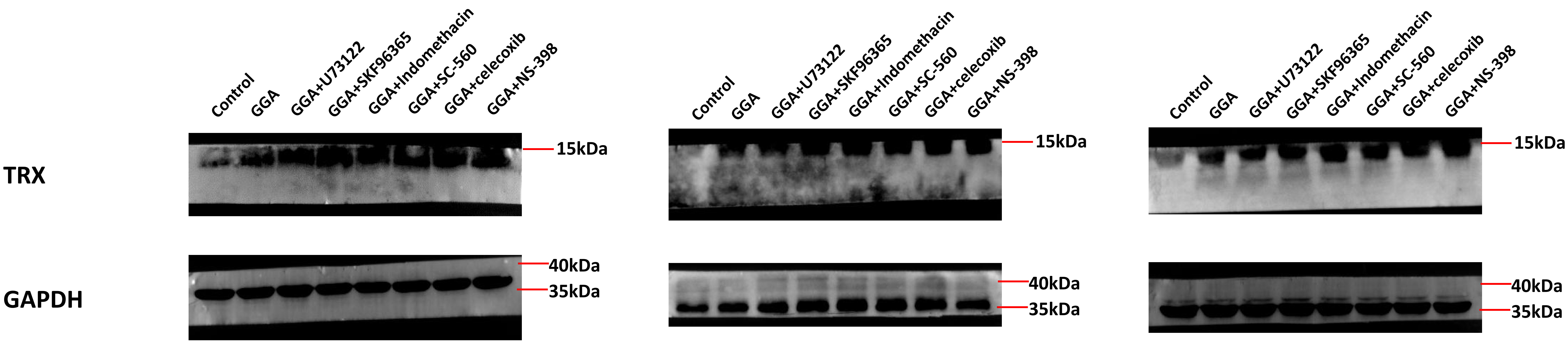

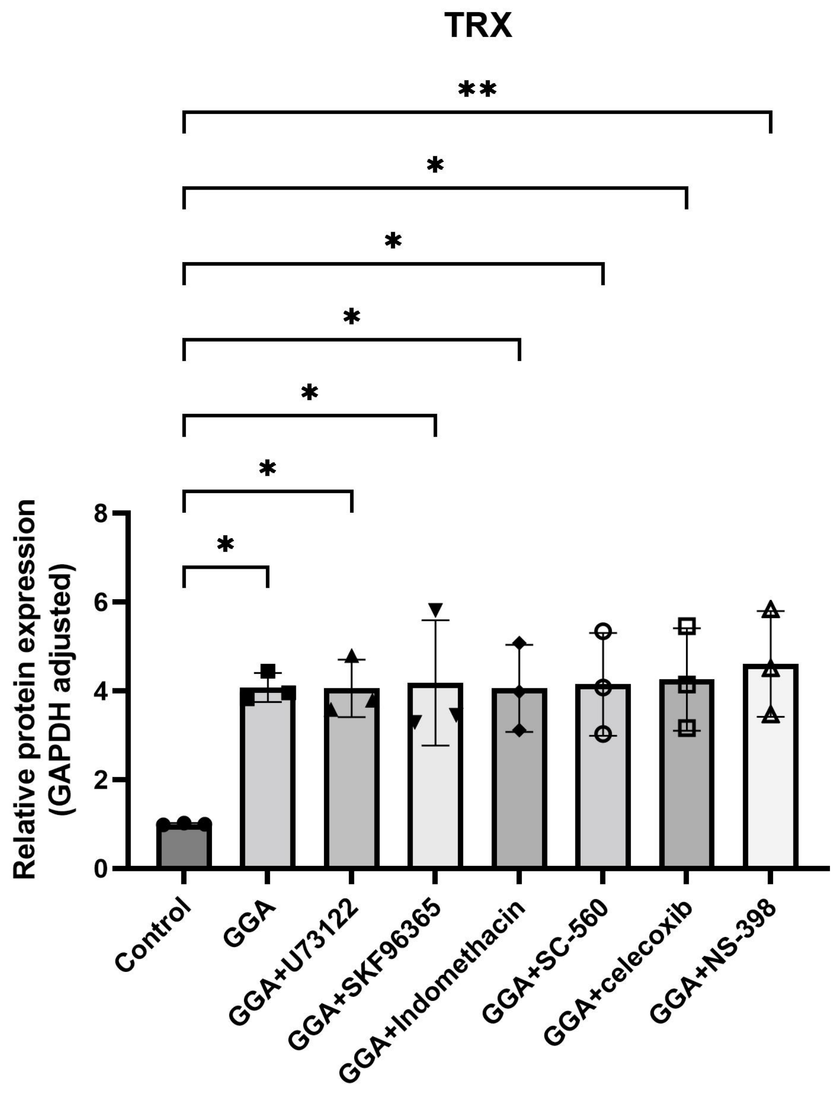

U-73122, SKF96365, Indomethacin, SC-560, celecoxib and NS-398 did not significantly inhibit GGA-induced TRX expression in PAM212 cells. The protein expression of TRX was detected by Western blot at 24 h post-stimulation. The relative expression of TRX was quantified and normalized to GAPDH. Treatments of 10 μM U-73122 (phospholipase C inhibitor), 20 μM SKF96365 (TRPC inhibitor), 150 μM Indomethacin (COX1/2 inhibitor), 0.3 μM SC-560 (COX1 inhibitor), 1 μM celecoxib (COX2 inhibitor) and 60 μM NS-398((COX2 inhibitor)) failed to remarkably inhibit the expression of TRX compared to the GGA-treated group. All inhibitors were purchased from MedChemExpress(China). ; values are mean ± SD of three independent experiments (*p<0.05, **p<0.01; one-way ANOVA with post-hoc Tukey´s).
